# A Rare Endobronchial Lipoma of the Right Middle Lobe Causing Middle Lobe Syndrome and Recurrent Post‐Obstructive Infection in a Patient With Interstitial Lung Disease, Managed by Lung‐Sparing Bronchoscopy

**DOI:** 10.1002/rcr2.70800

**Published:** 2026-11-02

**Authors:** Anastasios Palamidas, Kostandinos Papatheodosiou, Evagelia Karagianni, Fotios Daglis, Demosthenes Bouros

**Affiliations:** ^1^ Pneumonology Department Athens Medical Center Athens Greece; ^2^ 5th Pneumonology Department Sotiria Chest Diseases Hospital Athens Greece; ^3^ Pathology Department Athens Medical Center Athens Greece

**Keywords:** bronchoscopy, endobronchial lipoma, interstitial lung disease, middle lobe syndrome, obstructive pneumonia

## Abstract

Endobronchial lipomas are rare benign tumours that can cause airway obstruction and recurrent infection. A 70‐year‐old smoker with combined pulmonary fibrosis and emphysema presented with recurrent right middle lobe infections and declining pulmonary function. Computed tomography revealed a fat‐density lesion obstructing the middle lobe bronchus, with atelectasis and consolidation. Bronchoscopy and histology confirmed lipoma. Given limited respiratory reserve, lung‐sparing flexible bronchoscopy with snare electrocautery and argon plasma coagulation restored airway patency; retained purulent secretions were drained and infection treated. At 2 months, the patient was asymptomatic with radiological resolution; FVC improved from 75% to 83% predicted and DLCO from 35% to 50% predicted. Recovery followed concurrent recanalization, drainage and antimicrobial treatment, whose individual contributions cannot be separated. This case highlights bronchoscopic diagnosis and treatment of benign obstruction in persistent middle lobe disease. Longer surveillance is needed to assess durability.

## Graphical abstract

A 70‐year‐old smoker with combined pulmonary fibrosis and emphysema presented with recurrent right middle lobe infections and declining pulmonary function. This case describes bronchoscopic diagnosis of endobronchial lipoma and treatment of benign obstruction in persistent middle lobe disease, highlighting the need for longer surveillance to assess durability.

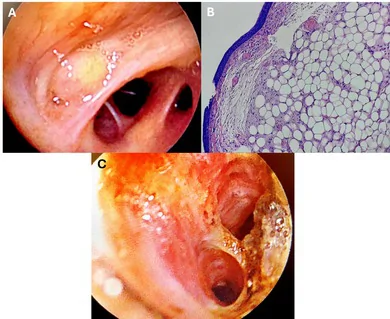

## 1 Introduction

Endobronchial lipomas are rare benign tumours arising from adipose tissue within the bronchial submucosa, accounting for approximately 0.1%–0.5% of pulmonary neoplasms [1, 2]. Despite their benign histology, endobronchial lipomas may cause progressive airway obstruction and distal lung damage [2, 3]. However, the course of pulmonary function following treatment of endobronchial obstruction and associated infection—particularly in patients with underlying interstitial lung disease, whose limited reserve may amplify the impact of superimposed obstruction—remains undocumented.

These lesions typically occur in the proximal tracheobronchial tree and most commonly involve the main or lobar bronchi [3]. Involvement of the right middle lobe bronchus is exceptionally rare, with fewer than 10 cases reported in the English literature [4, 5], and the clinical significance of middle lobe obstruction, particularly in patients with pre‐existing ILD, has not been systematically evaluated. High‐resolution computed tomography (HRCT) may suggest a diagnosis based on fat attenuation; however, interpretation may be difficult in the presence of post‐obstructive consolidation [5]. Bronchoscopy remains essential for definitive diagnosis and enables minimally invasive treatment of this condition [6].

The management of such lesions is particularly challenging in patients with interstitial lung disease, in whom limited respiratory reserve makes surgical resection high‐risk. We describe a patient with combined pulmonary fibrosis and emphysema (CPFE) [7] in whom a lung‐sparing bronchoscopic approach relieved an obstructing right middle lobe lipoma, with subsequent improvement in pulmonary function following recanalization and concurrent treatment of post‐obstructive infection, avoiding the need for surgery. A review of published case reports [1, 2] reveals that while bronchoscopic management of endobronchial lipomas has been described, none have documented serial pulmonary function testing before and after intervention in patients with interstitial lung disease, leaving the functional course after treatment of post‐obstructive disease in this high‐risk population uncertain.

## 2 Case Report

A 70‐year‐old male smoker (40 pack‐years) presented with a 7‐day history of cough, purulent sputum, fever, and right‐sided pleuritic chest pain. His medical history included obesity (body mass index > 38), arterial hypertension, prediabetes, coronary artery disease, and interstitial lung disease. The ILD was characterized as combined pulmonary fibrosis and emphysema (CPFE): HRCT showed upper‐lobe emphysema together with basal‐predominant reticulation, traction bronchiectasis, and honeycombing, consistent with a definite usual interstitial pneumonia (UIP) pattern [8]. The diagnosis was made radiologically; lung biopsy was not pursued, as the imaging pattern was considered diagnostic. The patient was followed for CPFE at another institution. His treating physician performed pulmonary function testing (PFT) approximately every 2–3 months to monitor functional status, with HRCT annually, as an individualized follow‐up plan. Complete longitudinal PFT and CT records were unavailable, so interval physiological and radiological stability could not be independently confirmed. He presented to our service specifically because of the acute post‐obstructive episode described below.

Earlier historical PFT reports were also available: spirometry on 16 March 2022 and a separate diffusion assessment on 14 April 2022. Both assessments preceded the later pre‐event reference assessment described below. The spirometry report recorded FVC 3.00 L (97% predicted), FEV1 2.32 L (102% predicted), and FEV1/FVC 77.3%. The subsequent diffusion report recorded haemoglobin‐corrected DLCO 12.16 mL/min/mmHg (58% predicted). A diffusion measurement obtained in March was technically uninterpretable and was excluded. These measurements provide an additional pre‐event reference point; percent‐predicted values are reported as calculated by the performing laboratory.

A later pre‐event reference assessment (approximately 3–6 months before bronchoscopy, when the patient was already clinically unwell with recurrent respiratory infections) showed FVC 86% predicted and DLCO 49% predicted (corrected for haemoglobin). During the year preceding the index admission, he experienced recurrent respiratory infections localizing to the right middle lung field. In the preceding 6 months, he had two exacerbations attributable to post‐obstructive infection, each requiring antibiotics, systemic corticosteroids, and supplemental oxygen, one of which required hospitalization; the current admission represented his second hospitalization. Repeat testing over this period showed a decline to FVC 75% predicted and DLCO 35% predicted. On examination, crackles and rhonchi were noted over the right middle lung field, together with bilateral basal Velcro‐like crackles.

Notably, endobronchial obstruction was not recognized on imaging during the previous hospitalization, when the appearances were attributed to post‐obstructive consolidation alone—a recognized diagnostic pitfall—which contributed to a delay in diagnosis. On the present admission, HRCT (Figure 1) demonstrated a fat‐density lesion (attenuation of approximately −100 HU) within the right middle lobe bronchus causing complete obstruction, with post‐obstructive atelectasis and consolidation consistent with middle lobe syndrome [5, 9]. Flexible bronchoscopy revealed a smooth, yellowish‐brown, polypoid, soft, and avascular lesion causing complete luminal occlusion at the carina between RB4 and RB5 (Figure 2A).

**FIGURE 1:**
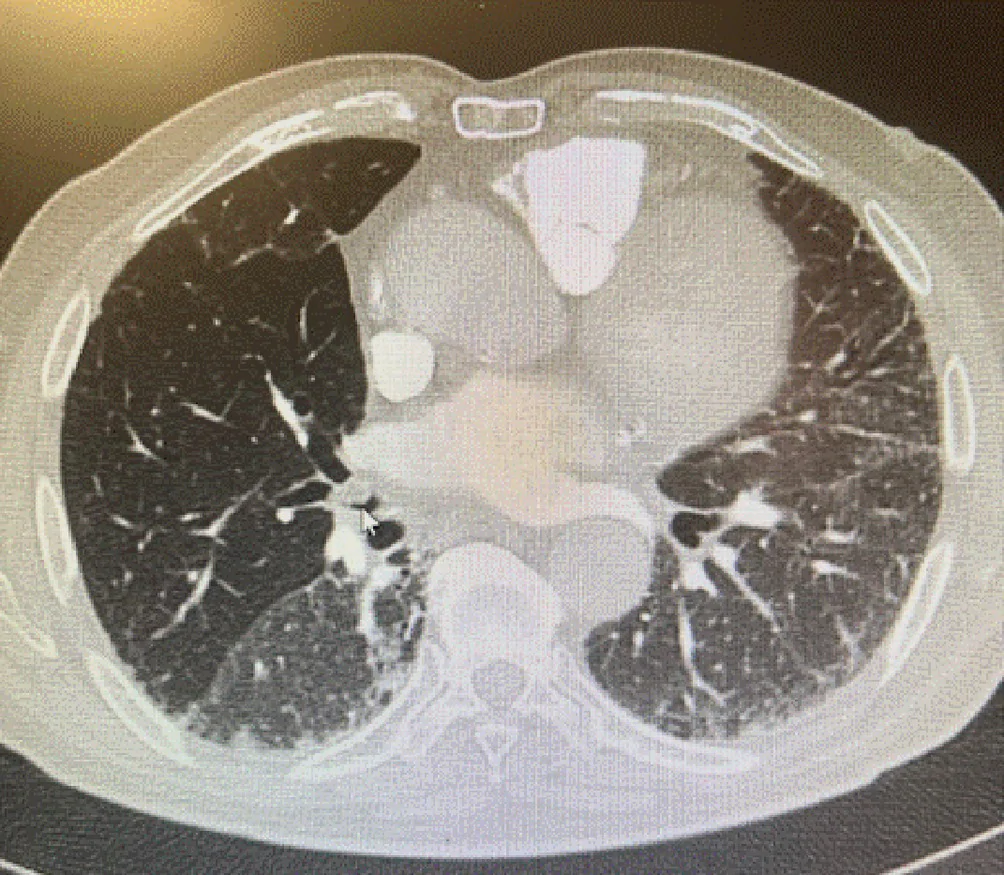
HRCT of the chest. The arrow indicates total luminal obstruction of the right middle lobe bronchus at the level of the carina between RB4 and RB5, with an endobronchial lesion of fat attenuation (approximately −100 HU), post‐obstructive atelectasis, and consolidation.

**FIGURE 2:**
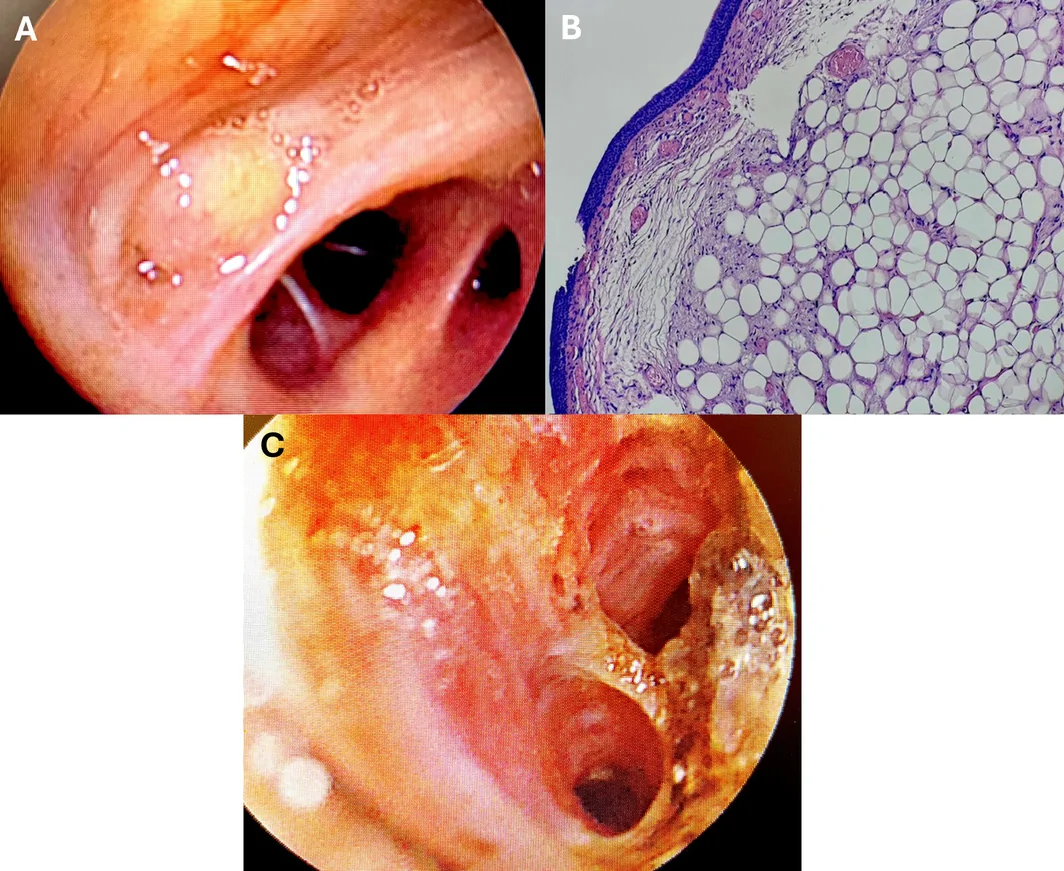
(A) Bronchoscopic image: A smooth, yellowish, polypoid, soft, avascular mass that obliterates the middle lobe orifice and causes total occlusion. (B) Histology: Mature adipocytes without cytological atypia or lipoblasts, covered by normal columnar respiratory epithelial lining (findings excluding well‐differentiated liposarcoma). (C) Restoration of complete airway patency after snare electrocautery debulking and argon plasma coagulation of the residual tissue. RB4 and RB5 were visible without residual lesions.

Initial forceps biopsy was performed. Histopathological examination confirmed a lipoma composed of mature adipocytes covered by a normal columnar respiratory epithelial lining, with no cytological atypia and no lipoblasts, thereby excluding well‐differentiated liposarcoma (Figure 2B).

Therapeutic bronchoscopy was performed as a flexible procedure under general anaesthesia via a laryngeal mask airway. Flexible bronchoscopy was chosen over rigid bronchoscopy because the lesion was distal (RB4/RB5 carina), soft, and avascular on initial inspection, making it amenable to flexible instrumentation; the laryngeal mask airway allowed adequate ventilation while preserving access for the flexible scope and instruments. The bulk of the lesion was resected with an electrocautery snare (ERBE VIO 200D; EndoCut mode, 40 W, effect 2, cut duration 2), and the residual broad base was ablated with argon plasma coagulation (ERBE VIO 200D; 40 W, 1 L·min−1). Mild, self‐limiting haemorrhage occurred at the base following APC and was controlled with suction alone, without any specific haemostatic intervention. After resection, copious purulent secretions were aspirated from the airway distal to the obstruction and sent for microbiological analysis, which grew Pseudomonas species and anaerobes (Figure 2). The procedure was completed without significant complications.

Complete airway patency was achieved. At the 2‐month follow‐up, chosen to allow resolution of post‐obstructive inflammation and consolidation while remaining within the patient's established ILD surveillance schedule (pulmonary function approximately every 2–3 months), the patient was asymptomatic, with radiological resolution of the obstruction (Figure 3) and pulmonary function approaching the later pre‐event reference values: FVC rose from 75% to 83% predicted, and DLCO from 35% to 50% predicted. This improvement followed recanalization, drainage of retained secretions, and concurrent treatment of infection. Base ablation was intended to minimize recurrence risk. He continued his established ILD surveillance (pulmonary function approximately every 2–3 months and HRCT annually) at the referring centre, with additional bronchoscopic and radiological review planned to monitor for recurrence (Table 1).

**FIGURE 3:**
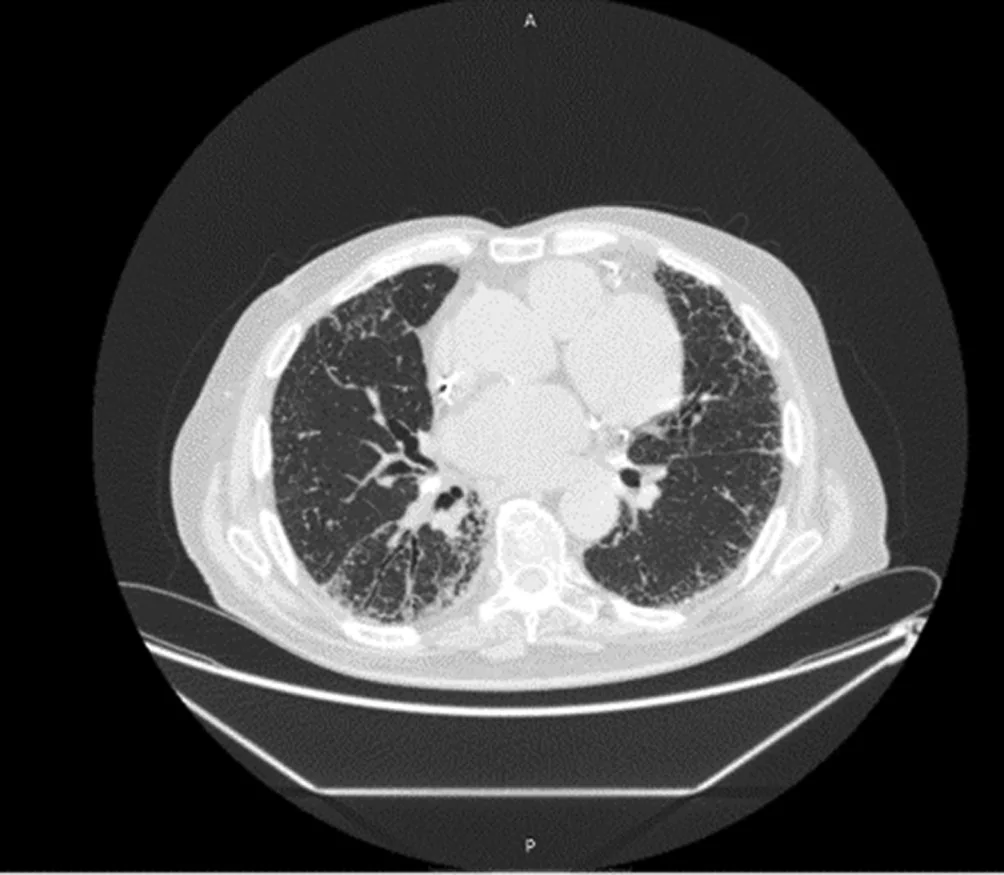
Post‐therapeutic CT scan 2 months after the procedure showing complete patency of the right middle lobe.

**TABLE 1:** Summary of the clinical course.

Time point	Event
Earlier historical testing	Spirometry on 16 March 2022: FVC 3.00 L (97% predicted), FEV1 2.32 L (102% predicted), and FEV1/FVC 77.3%. Separate diffusion assessment on 14 April 2022: haemoglobin‐corrected DLCO 12.16 mL/min/mmHg (58% predicted).
Later pre‐event reference assessment (approximately 3–6 months before bronchoscopy)	CPFE under follow‐up at another institution (pulmonary function approximately every 2–3 months and HRCT annually, according to the treating physician's individualized plan). Pulmonary function: FVC 86% predicted and haemoglobin‐corrected DLCO 49% predicted.
Preceding ~12 months	Recurrent right middle lobe infections; in the last 6 months, two exacerbations requiring antibiotics, systemic corticosteroids, and supplemental oxygen, with one hospitalization (the obstructing lesion was not recognized on imaging at that time). Progressive functional decline; pulmonary function fell to FVC 75% predicted, and DLCO 35% predicted.
Day 0 (second hospitalization)	7‐day history of cough, purulent sputum, fever, and right‐sided pleuritic chest pain.
Day 0	HRCT: fat‐density lesion in the right middle lobe bronchus with complete obstruction, post‐obstructive atelectasis, and consolidation (middle lobe syndrome).
Day 1	Diagnostic bronchoscopy revealed a smooth, yellowish, polypoid lesion occluding the RB4/RB5 orifice; forceps biopsy confirmed lipoma.
Day 2	Therapeutic bronchoscopy: snare electrocautery debulking and APC ablation of the base; copious purulent secretions were aspirated and cultured (Pseudomonas species and anaerobes). Antibiotics were administered, and the patient was discharged 7 days after the procedure.
2‐month follow‐up	Asymptomatic, with radiological resolution of the obstruction and improved pulmonary function following recanalization and treatment of post‐obstructive infection: FVC 83% predicted and DLCO 50% predicted, approaching the later pre‐event reference values.

## 3 Discussion

Endobronchial lipomas are uncommon benign tumours that may clinically and radiologically mimic malignant airway obstruction [2]. Despite their indolent nature, delayed diagnosis can result in recurrent, persistent post‐obstructive infection, leading to airway disease, such as bronchiectasis, and irreversible parenchymal damage [3]. They typically arise in large bronchi, where adipose tissue is present [3]. Smoking and obesity have been suggested as potential risk factors [1, 2]; our patient had both features (40 pack‐years; BMI > 38), although their contribution to lipoma formation in this case remains speculative.

In this patient, complete obstruction of the middle lobe bronchus produced middle lobe syndrome—post‐obstructive atelectasis, consolidation, and recurrent infection—rather than an acute exacerbation of interstitial lung disease in the ATS/ERS sense. The underlying ILD (CPFE) was the substrate that limited his respiratory reserve, whereas the lipoma was a treatable mechanical cause of airway obstruction associated with recurrent infection and functional decline. The microbiology supports this interpretation: growth of Pseudomonas species and anaerobes, together with copious purulent secretions aspirated distal to the obstruction, is characteristic of chronic infection trapped behind an obstructing lesion rather than typical community‐acquired pneumonia [10].

Serial pulmonary function measurements demonstrated deterioration during the obstructive/infective period and improvement after treatment. From the later pre‐event reference values of FVC 86% and DLCO 49% predicted, measurements fell to FVC 75% and DLCO 35% during the obstructive period, then increased to FVC 83% and DLCO 50% at 2‐month follow‐up. The absolute increases were 8 and 15 percentage points, respectively. This temporal pattern supports a potentially reversible component associated with the post‐obstructive episode but does not establish that bronchoscopic intervention alone caused the improvement or exclude fluctuation or progression of the underlying ILD. The interval decline between the 2022 historical assessments and the later pre‐event reference assessment (FVC 97% to 86% predicted; DLCO 58% to 49% predicted) may partly reflect the evolving obstruction and recurrent infection, as the patient was clinically unwell during the months preceding the index admission; however, progression of the underlying ILD over this interval cannot be excluded from the available measurements.

Bronchoscopic resection directly reopened the mechanically occluded bronchus and restored airway patency, allowing drainage of retained purulent secretions. We regard recanalization as the central anatomical effect of treatment and a plausible important contributor to recovery. This clinical interpretation is distinct from demonstrating a predominant effect on PFTs. Suction and drainage, antibiotic and supportive treatment, resolution of infection and atelectasis, and ordinary measurement variability, particularly for DLCO, may all have contributed; their individual effects cannot be quantified in this case. The available reference measurements helped assess deterioration and recovery, but this report does not establish that the surveillance frequency shortened the diagnostic delay.

Although benign, endobronchial lipomas can mimic malignancy; histological confirmation of mature adipocytes without atypia or lipoblasts is important to exclude a well‐differentiated liposarcoma, particularly in smokers. Complete treatment of the lesion base is essential because these tumours may recur if the base is inadequately ablated; in our patient, the broad base was ablated with argon plasma coagulation until the entire base showed white coagulative necrosis with no residual yellow adipose tissue visible, and the surrounding mucosa was intact; surveillance is planned to monitor for recurrence, as completeness of ablation cannot be histologically verified in situ. CT imaging plays an important role, particularly when fat attenuation is identified within an endobronchial lesion [5]; however, bronchoscopy remains the gold standard, allowing direct visualization, histological confirmation, and therapeutic intervention [6]. In our patient, the lesion was initially overlooked on imaging during an earlier admission, when the appearance was attributed to post‐obstructive consolidation alone—a recognized pitfall, since the fat attenuation of an endobronchial lipoma may be masked by adjacent consolidation [5]. This reinforces the value of maintaining a low threshold for bronchoscopy in persistent or recurrent lobar disease. Although pulmonary function approached the later pre‐event reference values after treatment, it is plausible that earlier intervention could further limit distal damage (e.g., bronchiectasis, irreversible fibrosis) in cases where obstruction persists longer or infection is more severe—a hypothesis that warrants prospective evaluation.

This case documents restoration of bronchial patency and subsequent improvement in pulmonary function following bronchoscopic treatment of an obstructing endobronchial lipoma and concurrent management of post‐obstructive infection in a patient with ILD (CPFE). FVC increased from 75% to 83% predicted and DLCO from 35% to 50% predicted at the 2‐month follow‐up. The temporal pattern is consistent with a reversible superimposed component but does not isolate the recanalization‐specific effect. Endoscopic resection with snare electrocautery and argon plasma coagulation of the base achieved airway reopening without surgical resection, a lung‐sparing outcome in a patient with limited respiratory reserve [6, 11]. Given that right middle lobe involvement is rarely reported in the literature (reference [4] describes one such case), this case underscores the importance of considering benign endobronchial lesions, such as lipomas, in persistent middle lobe disease and highlights the role of bronchoscopic diagnosis and treatment in selected patients with ILD who are unfit for surgery. Longer follow‐up and additional cases are required to assess durability and generalizability.

This report is subject to several limitations. First, the follow‐up period was short (2 months). Although recurrence rates for endobronchial lipomas after bronchoscopic resection are not well‐characterized in the literature, incomplete base ablation could predispose to late recurrence, which may occur months to years after intervention; longer surveillance (e.g., 12–24 months with serial bronchoscopy and imaging) is required to confirm durability. Second, the completeness of base ablation was assessed visually but not histologically verified. Third, bronchoscopic recanalization, suction and drainage of retained secretions, antibiotic and supportive treatment, and resolution of infection and atelectasis occurred over the same clinical period. Ordinary PFT variability, particularly for DLCO, may also have contributed. Their relative effects cannot be separated, and the observed improvement does not demonstrate that recanalization was the predominant cause of functional recovery. Fourth, pre‐event pulmonary function data were limited to the earlier historical reports and the later pre‐event reference assessment, and complete external PFT and CT records were not available; the interval decline between these assessments may reflect the evolving obstruction, progression of the underlying ILD, or both. Finally, as a single case, generalizability is uncertain across multiple dimensions: (1) ILD subtype—CPFE may respond differently to post‐obstructive relief than other ILD forms (e.g., nonspecific interstitial pneumonia, hypersensitivity pneumonitis); (2) lesion characteristics—our patient's lipoma was soft, avascular, and amenable to snare resection, whereas fibrous or vascular lesions may require alternative techniques; (3) patient factors—our patient's preserved FVC (75% predicted) may have facilitated recovery, whereas patients with more severe restriction may not tolerate the procedure or may have less reversible decline. Multi‐case series stratified by these factors are needed to define the boundaries of applicability.

## Author Contributions

**Anastasios Palamidas:** conceptualization, investigation, writing – original draft, methodology, validation, visualization, writing – review and editing, software, formal analysis, project administration, data curation, supervision, resources. **Kostandinos Papatheodosiou:** investigation, writing – original draft, writing – review and editing, validation, data curation, formal analysis. **Evagelia Karagianni:** writing – review and editing, writing – original draft, data curation, formal analysis. **Fotios Daglis:** data curation, formal analysis, writing – review and editing, writing – original draft. **Demosthenes Bouros:** conceptualization, investigation, writing – original draft, methodology, validation, visualization, writing – review and editing, formal analysis, project administration, data curation, supervision, resources.

## Funding

The authors have nothing to report.

## Ethics Statement

The authors certify that all aspects of this case report were carried out in strict adherence to the relevant national regulations, institutional policies, and ethical standards in Greece. The conduct of this work complies with the principles set forth in the Declaration of Helsinki.

## Consent

The authors declare that written informed consent was obtained from the patient for publication of this manuscript and accompanying images using the form provided by the Journal.

## Conflicts of Interest

The authors declare no conflicts of interest.

## Data Availability

Data sharing not applicable to this article as no datasets were generated or analysed during the current study.

## References

[1] E. M. Griffeth , B. A. Whitson , D. R. Huard , and B. R. Brown , “Endobronchial Lipoma: Case Report and Literature Review,” American Journal of the Medical Sciences 361, no. 5 (2021): 643–648. 10.1016/j.amjms.2020.07.010 32807376

[2] P. Akella , V. Jindal , B. S. Bhandari , and A. D. Siddiqui , “Meta‐Analysis of a Master Mimicker: Endobronchial Lipoma,” Chinese Clinical Oncology 9, no. 2 (2020): 11. 10.21037/cco.2019.08.17 31594363

[3] M. Muraoka , T. Oka , S. Akamine , et al., “Endobronchial Lipoma: Review of 64 Cases Reported in Japan,” Chest 123, no. 1 (2003): 293–296. 10.1378/chest.123.1.293 12527636

[4] A. Basoglu , B. Celik , A. O. Akdag , and A. T. Sengul , “Endobronchial Lipoma: A Rare Cause of Bronchial Occlusion,” Interactive Cardiovascular and Thoracic Surgery 3, no. 2 (2004): 263–264. 10.1016/j.icvts.2003.11.014 17670231

[5] D. Cao , Y. Sun , and S. Yang , “Endobronchial Lipoma: An Unusual Cause of Bronchial Obstruction,” Case Reports in Medicine 2011 (2011): 939808. 10.1155/2011/939808 PMC3182831 21969834

[6] L. Anbazhakan , A. Ullah , R. Munagala , et al., “Endobronchial Lipoma,” Autopsy & Case Reports 12 (2022): e2021377. 10.4322/acr.2021.377 PMC9083800 35574045

[7] V. Cottin , H. Nunes , P. Y. Brillet , et al., “Combined Pulmonary Fibrosis and Emphysema: A Distinct Underrecognised Entity,” European Respiratory Journal 26, no. 4 (2005): 586–593. 10.1183/09031936.05.00021005 16204587

[8] G. Raghu , M. Remy‐Jardin , L. Richeldi , et al., “Idiopathic Pulmonary Fibrosis (An Update) and Progressive Pulmonary Fibrosis in Adults: An Official ATS/ERS/JRS/ALAT Clinical Practice Guideline,” American Journal of Respiratory and Critical Care Medicine 205, no. 9 (2022): e18–e47. 10.1164/rccm.202202-0399ST PMC9851481 35486072

[9] T. Gudbjartsson and G. Gudmundsson , “Middle Lobe Syndrome: A Review of Clinicopathological Features, Diagnosis and Treatment,” Respiration 84, no. 1 (2012): 80–86. 10.1159/000336238 22377566

[10] M. S. Abers , B. P. Sandvall , R. Sampath , et al., “Postobstructive Pneumonia: An Underdescribed Syndrome,” Clinical Infectious Diseases 62, no. 8 (2016): 957–961. 10.1093/cid/civ1212 PMC4803103 26908806

[11] S. Bao , Y. Chen , C. Zhong , J. Zhang , and H. Tang , “Endobronchial Lipoma: A Rare Cause of Bronchial Obstruction—Case Report and Literature Review,” Discover Oncology 16 (2025): 1042. 10.1007/s12672-025-02856-x PMC12149369 40490664

